# IE63-specific T-cell responses associate with control of subclinical varicella zoster virus reactivation in individuals with malignancies

**DOI:** 10.1038/sj.bjc.6605542

**Published:** 2010-01-19

**Authors:** G N Malavige, L T Rohanachandra, L Jones, L Crack, M Perera, N Fernando, D Guruge, G S Ogg

**Affiliations:** 1 Department of Microbiology, Faculty of Medical Sciences, University of Sri Jayawardanapura, Nugegoda, Sri Lanka; 2 MRC Human Immunology Unit, Weatherall Institute of Molecular Medicine, Oxford NIHR Biomedical Research Centre and University of Oxford, Oxford, UK; 3 National Cancer Institute, Maharagama, Sri Lanka; 4 Department of Dermatology, Churchill Hospital, Oxford, UK

**Keywords:** varicella zoster virus, malignancy, T-cell responses, IE63

## Abstract

**Background:**

Reactivation of the varicella zoster virus (VZV) is more common in patients with malignancies; however, the molecular and cellular mechanisms underlying this susceptibility are unclear.

**Methods:**

Using *ex vivo* interferon-*γ* ELISpot assays, we set out to analyse VZV-specific immune responses in a large cohort of patients with malignancies.

**Results:**

We observed that patients with malignancies had impaired VZV-specific T-cell responses, particularly in those with haematological malignancies and breast carcinoma. Immediate-early protein 63 (IE63)-specific T-cell responses were significantly impaired in those with subclinical VZV re-activation.

**Conclusions:**

Our results suggest that T-cell responses to IE63 are important in controlling VZV replication.

Malignancy is associated with reactivation of chronic persistent viruses such as varicella zoster virus (VZV) (Whiteside, 2006), causing significant morbidity and mortality (Wade, 2006). Herpes zoster is more common in patients with malignancies (Schmader, 2001; Sorensen *et al*, 2004) and may lead to severe disease with multi-dermatomal involvement and visceral dissemination, which can be lethal (Gallagher and Merigan, 1979; Onunu and Uhunmwangho, 2004; Hackanson *et al*, 2005; Graue *et al*, 2006). However, apart from clinically apparent VZV reactivation, subclinical reactivation has also been reported in both immunocompetent and immunosuppressed individuals (Schunemann *et al*, 1998; Quinlivan *et al*, 2007). Although the mechanisms underlying such reactivation are unclear, it is thought that cell-mediated immune responses are vital in controlling VZV replication (Malavige *et al*, 2007, 2008b).

VZV glycoproteins I and E, immediate-early protein 63 (IE63) and four specific CD4+ T cells have been shown to circulate at persistently high frequencies in the peripheral blood of healthy seropositive donors without a history of reactivation (Jones *et al*, 2006, 2007; Malavige *et al*, 2007, 2008a). However, VZV-specific T-cell responses have been shown to be lower in elderly individuals and in patients with disease such as systemic lupus erythematosus (Miller, 1980; Levin *et al*, 2003; Park *et al*, 2004). As these groups of individuals are at a higher risk of herpes zoster, it seems that VZV-specific T cells are important in preventing virus reactivation. Some important studies conducted earlier have indeed shown that suppression of VZV-specific cellular immunity preceded the occurrence of herpes zoster (Arvin *et al*, 1978, 1980). However, with a more detailed understanding of VZV protein-specific responses, we can now study the mechanisms that are important in preventing virus reactivation. Therefore, we set out to analyse the overall VZV-specific immune responses and the immune responses to VZV IE63 and gE proteins, in a cohort of patients with malignancies to identify the associations with viral reactivation.

## Materials and methods

Fresh heparinised venous blood samples were obtained from 106 adult individuals with malignancies (before receiving chemotherapy) who were admitted to the Cancer Institute in Sri Lanka with a past history of primary VZV infection. Samples were also obtained from 26 patients after chemotherapy. Ethical approval for the study was obtained from the local ethics committee.

The mean age of the donors was 49.81 years (s.d.±17.5) and 55 (51.88%) were males. The mean age at which the patients had contracted primary VZV was 18 years (s.d.±12.67). In all, 88 patients (83.01%) had a solid malignancy (SM) and 18 (16.9%) had a haematological malignancy (HM) (Table 1). The healthy volunteers consisted of 19 healthy seropositive adults with a history of primary VZV infection but no clinical reactivation. Mean age of the seropositive donors was 34 years (range 25–62 years) with an average age of primary infection at 12.92 years (range 2–16 years).

*Ex vivo* ELISpot assays were performed as previously described (Smith *et al*, 2001), using freshly isolated PBMC and VZV antigen in the form of VZV vaccine (Varilrix, GSK, Rixensart, Belgium) or synthetic 20-mer peptides overlapping by 10 amino acids spanning the whole length of the gE or IE63 protein. PHA and FEC were included as positive controls and the plates were incubated overnight at 37 °C and 5% CO_2_. The ‘FEC’ peptides contain a panel of 23, 8–11 amino acid CD8+ T-cell epitopes of Epstein–Barr virus (EBV), Flu and CMV viruses and have been used for quality control in ELISpot assays (Currier *et al*, 2002).

Quantitative real-time PCR was performed as previously described using the ABI Prism 7700 sequence detector system (Foster City, CA, USA) (Pevenstein *et al*, 1999; Quinlivan *et al*, 2007). Forward primers (5′-CGTACACGTATTTTCAGTCCTCTTC-3′) and reverse primers (5′-GGCTTAGACGTGGAGTTGACA-3′) for VZV ORF29 and a probe (5′-(FAM)CCCGTGGAGCGCGTCGAAA(TAMRA)-3′) were used (Pevenstein *et al*, 1999). The real-time fluorescence values were measured by the quantity of a reporter dye FAM released during amplification. The threshold limit was set in the linear phase of exponential amplification after viewing the log-linear view of the amplification plot.

### Statistical analysis

Data analyses were carried out using GraphPad Prism 4 (San Diego, CA, USA). Data were analysed using nonparametric tests, as frequency distribution curves showed that the data were not normally distributed. Wilcoxon's matched-pairs test was used to determine the *P*-value in paired samples whereas the Mann–Whitney test was used to calculate the *P*-values in unpaired data. Spearman's correlation coefficient was used to determine the correlation and the *P*-value when determining correlations between the absolute white cell count and VZV-specific T-cell responses and absolute lymphocyte count and VZV-specific T-cell responses.

## Results

### Overall *ex vivo* T-cell responses in patients with malignancies

**Figure 1:**
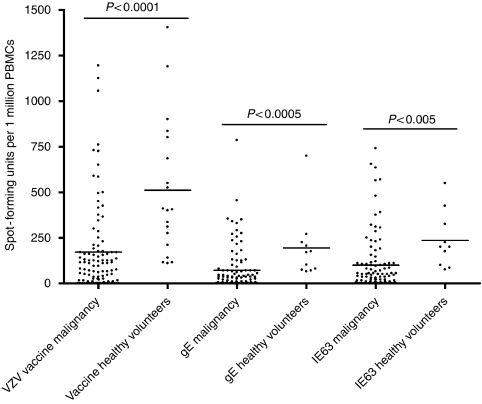
*Ex vivo* IFN*γ* ELISpot responses to VZV live attenuated vaccine, overlapping gE 20-mer peptide pool and overlapping IE63 20-mer peptide pools in patients with malignancies and healthy seropositive volunteers with no evidence of VZV reactivation.

**Figure 2:**
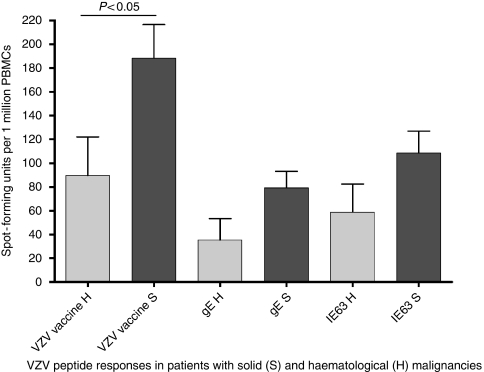
*Ex vivo* IFN*γ* ELISpot responses to VZV live attenuated vaccine, overlapping gE 20-mer peptide pool and overlapping IE63 20-mer peptide pool in patients with haematological and solid malignancies. The error bars represent the s.e.m.

We observed that overall VZV-specific T-cell responses were significantly diminished (*P*<0.0001) in patients with malignancies (mean 170.9, s.d.±244.3) when compared with healthy VZV seropositive individuals (mean 511.3, s.d.±372.3; Figure 1). Patients with haematological malignancies and breast cancer had the lowest VZV-specific T-cell responses. Those with haematological malignancies had significantly lower overall T-cell responses to VZV (as measured by responses to the VZV live attenuated vaccine) than those with solid malignancies (Table 1; *P*<0.05). However, there was no difference between the two groups for gE or IE63 overlapping 20-mer peptides (Figure 2). Among those with solid malignancies, patients with breast carcinoma had significantly lower VZV-specific T-cell responses (*P*<0.0001). The overall VZV-specific T-cell responses in individuals with different malignancies are shown in Table 1. Patients with haematological malignancies had significantly higher (*P*<0.05) total white cell count (mean 11 746, s.d.±9079) and significantly higher (*P*<0.005) lymphocyte count (mean 3986, s.d.±3986) than patients with solid malignancies. However, we found no correlation with the T-cell response with either total white cell or lymphocyte counts. We also observed no correlation between VZV-specific T-cell responses and age of the individual or time since occurrence of primary infection.

A significant difference was not observed between the pre- and post-chemotherapy total white cell or lymphocyte counts in the 26 patients for whom we had post-chemotherapy samples taken at 1–2 weeks (data not shown). Furthermore, we found that there were no significant differences in the overall VZV-specific functional T-cell responses or for responses to gE or IE63 overlapping peptide pools in this group of patients. VZV-specific T-cell responses also did not significantly differ between patients on different chemotherapy regimes.

### Subclinical viraemia in patients with malignancies

**Figure 3:**
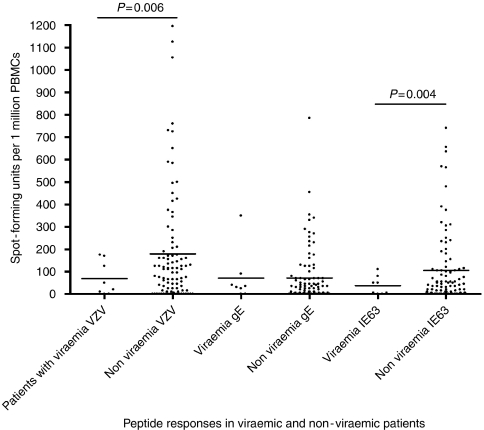
*Ex vivo* IFN*γ* ELISpot responses to VZV live attenuated vaccine (VZV), overlapping gE 20-mer peptide pool and overlapping IE63 20-mer peptide pool in patients with malignancies who had subclinical VZV viraemia and those who were non-viraemic.

Of the 106 patients recruited in our study, none had clinical evidence of VZV reactivation. However, VZV DNA was detected in eight (7.5%) patients. The viral loads in these patients varied from 30.93 to 198 viral copies per ml of blood (mean 108.18, s.d.±53.58). We observed no viral reactivation in the healthy controls. Overall, those who had subclinical VZV reactivation had significantly lower (*P*<0.01) overall VZV-specific T-cell responses (mean 68.75, s.d.±75.9) than those with no reactivation (mean 179.6, s.d.±251.8). Furthermore, responses to VZV IE63 overlapping 20-mer peptides were significantly lower (*P*<0.005) in those with subclinical reactivation (mean 37.5, s.d.±41.9) than in those with no reactivation (mean 105.2, s.d.±162.1). There was no difference in T-cell responses in these two groups for VZV gE overlapping 20-mer peptides (Figure 3).

## Discussion

We have shown that VZV-specific functional T-cell responses are significantly impaired in patients with malignancies when compared with healthy seropositive controls. These responses were lowest in those with haematological malignancies and breast carcinoma. It has been shown previously that patients with colorectal carcinomas did not have any significant reduction in CMV- and influenza-specific T-cell responses (Kiewe *et al*, 2009), assessed using MHC class I tetramers. However, as detection of antigen-specific cells by tetramers does not depend on T-cell function, it is not clear whether the antiviral T cells of these patients were functionally similar to healthy controls.

Subclinical VZV viraemia was observed in eight (7.5%) of the patients. Interestingly, overall mean VZV vaccine-specific T-cell responses, and responses to IE63 overlapping 20-mer peptides were significantly lower in those with VZV reactivation when compared with other patients, suggesting that IE63-specific T-cell responses could be important in the control of VZV replication. However, it will clearly be important to extend these findings to other viral proteins.

We failed to see any significant differences between VZV-specific T-cell responses in our patient cohort in both before and after chemotherapy. However, as post-chemotherapy samples were obtained 1–2 weeks after the initiation of chemotherapy, this might not suffice for the occurrence of immunosuppressive effects of the chemotherapy. Therefore, it is important to expand this study in a larger population of patients who received chemotherapy and who were followed up for longer periods.

In summary, we have shown that a large cohort of patients with malignancies had impaired T-cell responses to VZV and that virus-specific T-cell responses were lowest in those with subclinical virus reactivation. Interestingly, IE63-specific T-cell responses were significantly impaired in those with subclinical VZV reactivation, suggesting that T-cell responses to IE63 could be important in controlling VZV reactivation. Furthermore, the data suggest that enhancement of IE63-specific T-cell responses may prove beneficial in future vaccination strategy development.

## Figures and Tables

**Table 1:** The absolute white cell counts, the absolute lymphocyte counts and *ex vivo* VZV live attenuated vaccine-specific ELISpot responses in patients with malignancies before initiation of any chemotherapy

**Site of malignancy, *n* (%)**	**Absolute white cell count (mean±s.d.)**	**Absolute lymphocyte count (mean±s.d.)**	**T-cell responses to the live attenuated VZV vaccine (spot forming units per1 million PBMCs) (mean±s.d.)**
Haematological, 18 (16.9)	11 746±9079	10 929±24 737	89.72±137
Colon and rectal cancer, 13 (12.3)	6657±2499	1972±870.7	235±304.7
Ovary, 14 (13.2)	8014±3101	2353±1142	207.1±300.2
Uterus and cervix, 12 (11.3)	5414±2949	1559±538.8	172.1±346.3
Lung, 12 (11.3)	9254±3713	1837±1037	197.5±191.2
Osteosarcoma, 7 (6.6)	5786±2392	1626±1146	170.7±138.5
Breast, 7 (6.6)	9900±9787	2313±956.1	93.57±68.54

Abbreviations: PBMC=peripheral blood mononuclear cell; VZV=varicella zoster virus.
